# Resistance of *Stenotrophomonas maltophilia* to Fluoroquinolones: Prevalence in a University Hospital and Possible Mechanisms

**DOI:** 10.3390/ijerph120505177

**Published:** 2015-05-13

**Authors:** Wei Jia, Jiayuan Wang, Haotong Xu, Gang Li

**Affiliations:** 1 Medical Experimental Center, General Hospital of Ningxia Medical University, 804 Shengli Street, Yinchuan City, Ningxia Hui Autonomous Region 750004, China; E-Mail: gone.lee@163.com; 2 School of Laboratory Medicine, Ningxia Medical University, 1160 Shengli Street, Yinchuan City, Ningxia Hui Autonomous Region 750004, China; E-Mails: wjy10816170105@163.com (J.W.); xht304@126.com (H.X.)

**Keywords:** *Stenotrophomonas maltophilia*, antimicrobial resistance, reserpine, fluoroquinolone, pulsed-field gel electrophoresis

## Abstract

*Objective*: The purpose of this study was to investigate the clinical distribution and genotyping of *Stenotrophomonas maltophilia*, its resistance to antimicrobial agents, and the possible mechanisms of this drug resistance. *Methods*: *S. maltophilia* isolates were collected from clinical specimens in a university hospital in Northwestern China during the period between 2010 and 2012, and were identified to the species level with a fully automated microbiological system. Antimicrobial susceptibility testing was performed for *S. maltophilia* with the Kirby-Bauer disc diffusion method. The minimal inhibitory concentrations (MIC_s_) of norfloxacin, ofloxacin, chloramphenicol, minocycline, ceftazidime, levofloxacin and ciprofloxacin against *S. maltophilia* were assessed using the agar dilution method, and changes in the MIC of norfloxacin, ciprofloxacin and ofloxacin were observed after the addition of reserpine, an efflux pump inhibitor. Fluoroquinolone resistance genes were detected in *S. maltophilia* using a polymerase chain reaction (PCR) assay, and the expression of efflux pump *smeD* and *smeF* genes was determined using a quantitative fluorescent (QF)-PCR assay. Pulsed-field gel electrophoresis (PFGE) was employed to genotype identified *S. maltophilia* isolates. *Results*: A total of 426 *S. maltophilia* strains were isolated from the university hospital from 2010 to 2012, consisting of 10.1% of total non-fermentative bacteria. The prevalence of norfloxacin, ciprofloxacin and ofloxacin resistance was 32.4%, 21.9% and 13.2% in the 114 *S. maltophilia* isolates collected from 2012, respectively. Following reserpine treatment, 19 *S. maltophilia* isolates positive for efflux pump were identified, and high expression of *smeD* and *smeF* genes was detected in two resistant isolates. *gyrA*, *parC*, *smeD*, *smeE* and *smeF* genes were detected in all 114 *S. maltophilia* isolates, while *smqnr* gene was found in 25.4% of total isolates. Glu-Lys mutation (GAA-AAA) was detected at the 151th amino acid of the *gyrA* gene, while Gly-Arg mutation (GGC-CGC) was found at the 37th amino acid of the *parC* gene. However, no significant difference was observed in the prevalence of *gyrA* or *parC* mutation between fluoroquinolone-resistant and -susceptible isolates (*p*> 0.05). The *smqnr* gene showed 92% to 99% heterogenicity among the 14 *S. maltophilia* clinical isolates. PFGE of 29 *smqnr* gene-positive *S. maltophilia* clinical isolates revealed 25 PFGE genotypes and 28 subgenotypes. *Conclusions*: Monitoring the clinical distribution and antimicrobial resistance of *S. maltophilia* is of great significance for the clinical therapy of bacterial infections. Reserpine is effective to inhibit the active efflux of norfloxacin, ciprofloxacin and ofloxacin on *S. maltophilia* and reduce MIC of fluoroquinolones against the bacteria. The expression of efflux pump *smeD* and *smeF* genes correlates with the resistance of *S. maltophilia* to fluoroquinolones.

## 1.Introduction

*Stenotrophomonas maltophilia*, an aerobic, non-fermentative bacterium, is predominantly found in immunocompromised individuals and those receiving long-term, large-dose broad-spectrum antimicrobial agents [1,2,3]. This bacterium, which frequently colonizes medical devices, may induce catheter-associated infections, bacteriaemia, urinary infections, and infections at other multiple sites [4,5]. Among the non-fermentative bacteria, *S. maltophilia* ranks third to *Pseudomonas aeruginosa* and *Acinetobacter* spp. in the prevalence among clinical isolates [1]. The natural resistance to imipenem and high resistance to multiple clinically commonly used antimicrobial agents leave few antimicrobial options for this species, making treatment of patients infected with *S. maltophilia* very difficult [6,7,8]. Fluoroquinolones are a class of chemicals that are effective for the treatment of infections caused by *S. maltophilia* infections [9,10,11], however, there is an increasing reported prevalence of resistance to fluoroquinolones in *S. maltophilia* [12,13,14]. It is reported that the resistance of *S. maltophilia* to fluoroquinolones may be mainly caused by the mutation at the target sites of DNA gyrase and topoisomerase [15,16,17,18], plasmid or chromosome-mediated mutations of drug resistance genes [19,20] and drug efflux pumps [21,22,23]. Since multiple mechanisms are found to be involved in the development of resistance to fluoroquinolones in *S. maltophilia*, an increasing prevalence of fluoroquinolone resistance is detected in *S. maltophilia*. Understanding of the mechanisms underlying fluoroquinolone resistance may therefore be helpful to interrupt and prevent the emergence of antibiotic-resistant *S. maltophilia* and reduce the prevalence and mortality of *S. maltophilia* infections. In this study, we investigated the distribution and antimicrobial resistance of 426 clinical strains of *S. maltophilia* isolated during the period between 2010 and 2012from a university hospital located in Northwestern China, and explored the possible mechanisms of the observed resistance, so as to provide evidence as a basis for the inappropriate clinical use of antimicrobial agents and the control and prevention of *S. maltophilia* infections. In addition, pulsed-field gel electrophoresis (PFGE) was used for genotyping clinical isolates of *S. maltophilia* to understand the characteristics of hospital-acquired infections of this bacterium.

## 2. Materials and Methods

### 2.1. S. maltophilia Isolates

During the period from 2010 through 2012, a total of 426 clinical isolates of *S. maltophilia* were collected from various clinical specimens in a university hospital in Northwestern China. All isolateswere identified to species level with a VITEK-2 COMPACT fully automated microbiological system (BioMérieux, Inc.; Durham, NC, USA).

### 2.2. Determination of Minimum Inhibitory Concentration of Fluoroquinolones

The minimum inhibitory concentration (MIC) of seven fluoroquinolones including norfloxacin, ofloxacin, chloramphenicol, minocycline, ceftazidime, levofloxacin, and ciprofloxacin (National Institutes for Food and Drug Control, Beijing, China; all potency >95%), on *S. maltophilia* was estimated using the agar dilution method [24], and was assessed according to the Clinical Laboratory Standard Institute (CLSI) published interpretive criteria, while *Escherichia coli* ATCC25922 (Shanghai Harmony Biotechnology Co., Ltd.; Shanghai, China) served as quality control bacterial strains.

### 2.3. Effect of Reserpine on Fluoroquinolone MIC

The Mueller-Hinton (M-H) agar medium (Oxoid, Basingstoke, United Kingdom) containing norfloxacin, ciprofloxacin, and ofloxacin at a 1:2 dilution was added with an efflux pump inhibitor reserpine (Dalian Meilun Biology Technology Co., Ltd.; Dalian, China) at a final concentration of 20 mg/L to determine the MIC of *S. maltophilia* to these three fluoroquinolones in the presence of efflux pump inhibitor, while the medium treated with reserpineat the same concentration served as controls. The change in the MIC of norfloxacin, ciprofloxacin, and ofloxacin on *S. maltophilia* was evaluated before and after reserpine treatment, and a MIC reduction of 1/4 or less was considered efflux pump positive [25,26].

### 2.4. Detection of Fluoroquinolone Resistance Genes in S. maltophilia Clinical Isolates

Genomic DNA was isolated from *S. maltophilia* colonies using an Invitrogen™ genomic DNA extraction kit, and fluoroquinolone resistance genes were detected in the 114 clinical isolates of *S. maltophilia* using a polymerase chain reaction (PCR) assay with the primers (Table 1) synthesized by the Sangon Biotech (Shanghai) Co., Ltd. (Shanghai, China), including *qnrA*, *qnrB*, *qnrS*, *qnrC*, *qnrD*, *aac-lb-Cr*, *qepA*, *oqxA*, *oqxB*, *smeE*, *smeF*, *smeD*, *gyrA*, *parC* and *smqnr* [27,28,29,30]. PCR was performed with a 25 μL system containing1 μL template DNA, 1 μL of the forward and reverse primers, 12.5 μL Premix Taq (BioTeke Biotech Co., Ltd.; Beijing, China), and 9.5 μL ddH_2_O under the following conditions: pre-degeneration at 95 °C for 5 min, followed by 30 cycles of degeneration at 95 °C for 30 s, annealing at the temperature shown in Table 1 for 30 s, and extension at 72 °C for 1 min, and final extension at 72 °C for 7 min. The amplification products were checked on a 1.5% agarose gel by electrophoresis.

**Table 1:** PCR primer sequence and PCR product size.

Fluoroquinolone Resistance Gene	Sequence	Annealing Temperature (°C)	Product Size (bp)
*qnrA*	F: 5’-AGAGGATTTCTCACGCCAGG-3’; R: 5’-TGCCAGGCACAGATCTTGAC-3’	63	580
*qnrB*	F: 5’-GGMATHGAAATTCGCCACTG-3’; R: 5’- TTTGCYGYYCGCCAGTCGAA	56	264
*qnrS*	F: 5’-GCAAGTTCATTGAACAGGGT-3’; R: 5’-TCTAAACCGTCGAGTTCGGCG-3’	56	428
*qnrC*	F: 5’-GGGTTGTACATTTATTGAATCG-3’; R: 5’-CACCTACCCATTTATTTTCA-3’	56	310
*qnrD*	F: 5’-GGGTTGATTTAACTGATAC-3’; R: 5’-TTCGCACTTTTCTAATATGAC-3’	56	310
*aac-Ib-Cr*	F: 5’-TTGCGATGCTCTATGAGTGGCTA-3’; R: 5’-CTCGAATGCCTGGCGTGTTT-3’	58	482
*qepA*	F: 5’-GCAGGTCCAGCAGCGGGTAG-3’; R: 5’-CTTCCTGCCCGAGTATCGTG-3’	48	199
*oqxA*	F: 5’-CTTGCACTTAGTTAAGCGCC-3’; R: 5’-GAGGTTTTGATAGTGGAGGTAGG-3’	65	866
*oqxB*	F: 5’-GCGGTGCTGTCGATTTTA-3’; R: 5’- TACCGGAACCCATCTCGAT-3’	65	781
*smeE*	F: 5’-AGCTCGACGCCACGGTA-3’; R: 5’- TGGCCTGGATCGAGAGCA-3’	55	803
*smeF*	F: 5’-GCCACGCTGAAGACCTA-3’; R: 5’- CACCTTGTACAGGGTGA-3’	55	800
*smeD*	F: 5’-CCAAGAGCCTTTCCGTCAT-3’; R: 5’- TCTCGGACTTCAGCGTGAC-3’	58	150
*gyrA*	F: 5’-AACTCAACGCGCACAGCAACAAGCC-3’; R: 5’-CCAGTTCCTTTTCGTCGTAGTTGGG-3’	58	300
*parC*	F: 5’-ATCGGCGACGGCCTGAAGCC-3’; R: 5’-CGGGATTCGGTATAACGCAT-3’	55	273
*smqnr*	F: 5’-GCTCTAGAGCTCTACGAATGCGATTTCTCCG-3’; R: 5’- CGGAATTCCGAAACTGGCACCGCTCACG-3’	52	817
gyrA*	F: 5’-AACTCAACGCGCACAGCAACAAGCC-3’; R: 5’-CCAGTTCCTTTTCGTCGTAGTTGGG-3’	58	300
smeD*	F: 5’-CCAAGAGCCTTTCCGTCAT-3’; R: 5’- TCTCGGACTTCAGCGTGAC-3’	58	150
smeF*	F: 5’-CCAACGCGGATCGTGATATC-3’; R: 5’-TGCTCATCCAGGCTGACATTC-3’	58	100

* For quantitative fluorescence PCR.

Following electrophoresis for 30 min, the agarose gel was stained with ethidium bromide (0.5 μg/mL), and then visualized with a gel imaging analysis system. The target DNA fragment was sequenced by the Beijing Sunbiotech Co., Ltd. (Beijing, China), and aligned to the sequences released in GenBank.

### 2.5. Quantitative Fluorescent (QF)-PCR

A total of six *S. maltophilia* isolates were used for QF-PCR assay, including three randomly selected efflux pump-positive isolates that were resistant to fluoroquinolones, one randomly selected efflux pump-negative isolate that was resistant to fluoroquinolone, one efflux pump-negative isolate that was sensitive to norfloxacin, ciprofloxacin, and ofloxacin, and one *S. maltophilia* ATCC13637 isolate (Shanghai Harmony Biotechnology Co., Ltd.) that served as an efflux pump-negative control isolate. Total RNA was isolated from *S. maltophilia* isolates using the AxyPrep RNA extraction kit (Axygen Biosciences, Inc.; Union City, CA, USA) following the manufacturer’s instructions.

Total RNA was reversely transcribed into cDNA using a cDNA reverse transcription kit (Beijing TransGen Biotech Co., Ltd.; Beijing, China) according to the manufacturer’s instructions. Briefly, cDNA was synthesized in a 20 μL reaction system containing 1 μg RNA, 1 μL reverse primer, 10 μL 2 × TS Reaction Mix, 1 μL RT Enzyme Mix, 1 μL gDNA Remover and 7 μL RNase-free water under the following condition: at 25 °C for 10 min, at 42 °C for 30 min, and at 85 °C for 5 min. The mRNA expression of *smeD* and *smeF* genes in *S. maltophilia* isolates was detected using a QF-PCR assay with the primers (Table 1) synthesized by the Sangon Biotech (Shanghai) Co., Ltd. [31,32] on a Roche Light cycler 480 PCR System (Roche, Basel, Switzerland), and *gyrA* served as an internal reference gene. The PCR assays were performed by pre-denaturation for 30 s at 95 °C, followed by 45 cycles of 5 s at 95 °C and 30 s at 58 °C. Melting curve analysis was also used to check the specificity of the amplification reaction. ddH_2_O, as alternative of template cDNA, was used as a negative control. Relative quantity of *smeD* and *smeF mRNA* expression was calculated by using the 2^−ΔΔCT^ method, while the relative expression of *gyrA mRNA* in the *S. maltophilia* ATCC13637 isolate was defined as 1.

### 2.6. PFGE

PFGE was performed in 29 *S. maltophilia* clinical isolates that were positive for *smqnr* gene and one quality control isolate *Salmonella typhi* H9812. Briefly, single colony was added with 20 µL lysozyme (20 mg/mL; Beijing TransGen Biotech Co., Ltd.), placed in water bath at 55 °C for 15 min, and then added with 20 µL protease K (20 mg/mL; Beijing TransGen Biotech Co., Ltd.).Then, 400 µL agarose (1%) with a low melting point was added, mixed, and frozen. The mixture was transferred to 1 mL lysozyme buffer and 5 µL protease K (20 mg/mL), placed at a water bath at 55 °C for 2 h, and then the supernatant was discarded. The sediment was rinsed twice with pre-warmed (54 °C) pure water, and washed three times with pre-warmed (54 °C) 1 × TE buffer, of 10 min each time. The gel was cleaved with 100 µL restriction enzyme *Xba I* on a water batch at 37 °C for 2 h. Finally, electrophoresis was performed in 1 × TBE buffer at 14 °C, 6 V/cm, 120° angular field of view for 22 h. Pulse time was increased linearly from 5 s initially to 35 s finally during the run.Following electrophoresis for 30 min, the agarose gel was stained with ethidium bromide (0.5 μg/mL), and then visualized with a gel imaging analysis system. The target DNA fragment was sequenced by the Beijing Sunbiotech Co., Ltd. PFGE patterns were interpreted according to the criteria suggested by Tenover *et al.* [33].

### 2.7. Statistics

All data were processedin the software WHONET version 5.6, and all statistical analyses were done with the software SPSS version 11.5 (SPSS Inc.; Chicago, IL, USA). The differences of proportions were tested for statistical significance with chi-square test, while Student *t* test was employed to compare between groups. A *p* value <0.05 was considered statistically significant.

## 3. Results

### 3.1. Prevalence of S. maltophilia clinical Isolates

A total of 426 *S. maltophilia* strains were isolated from the university hospital from 2010 to 2012, consisting of 10.1% (426/4225) of total non-fermentative bacteria and 1.8% (426/23,880) of total clinical bacterial isolates, and the prevalence of *S. maltophilia* ranked third to *P. aeruginosa* and *A. baumannii* and sixth in all Gram-negative bacilli. Of the 426 *S. maltophilia* clinical isolates, 83.3% were isolated from sputum and throat swab, 4.5% from catheters andexcreta, 1.6% from blood, and 7.1% from cerebrospinal fluid, sterile body fluid and other samples.

### 3.2. Resistance of S. maltophilia to Fluoroquinolones

The 426 clinical isolates of *S. maltophilia* showed the highest susceptibility to minocycline, with a 0.5% prevalence of minocycline resistance detected, while 3.3% and 74.3% of the isolates were resistant to levofloxacin and sulfamethoxazole, respectively. During the study period from 2010 to 2012, no significant differences were detected in the prevalence of minocycline or levofloxacin resistance in *S. maltophilia* clinical isolates (all *p* values > 0.05); however, there was a significant rise seen in the prevalence of sulfamethoxazole resistance (χ^2^ = 36.963, *p* < 0.01). In addition, the prevalence of resistance to norfloxacin, ciprofloxacin, and ofloxacin appeared a rise tendency in *S. maltophilia* clinical isolates during the study period (Table 2).

### 3.3. Fluoroquinolone Resistance in S. maltophilia Isolated from Various Departments of the Hospital

The prevalence of fluoroquinolone resistance varied in *S. maltophilia* isolated from different departments of the hospital (Table 3). A higher prevalence (95%) of sulfamethoxazole resistance was detected in *S. maltophilia* isolated from the Department of Neurology than from the Intensive Care Unit (ICU), Department of Respiratory Medicine, and Department of Pediatrics (*p* = 0.026, 0.029 and 0.036, respectively), while the highest prevalence of levofloxacin resistance was found in other departments (*p* = 0.017). However, no significant difference was observed in the prevalence of minocycline resistance in *S. maltophilia* isolated from various departments of the hospital (*p* > 0.05).

**Table 2:** Resistance of *Stenotrophomonas maltophilia* to three fluoroquinolone antibacterial drugs from 2010 to 2012.

Fluoroquinolone Antibacterial	2010 (*n* = 69)		2011 (*n* = 148)		2012 (*n* = 209)		*χ^2^* value	*p* value
	Resistant (%)	Susceptible (%)	Resistant (%)	Susceptible (%)	Resistant (%)	Susceptible (%)		
Levofloxacin	0	100	4	94.6	4.1	93	3.102	0.212
Sulfamethoxazole	53	47	66	34	86.6	13.4	36.963	0
Minocycline	0	100	0.5	99	0.7	98	0.695	0.706

**Table 3:** Resistance to three fluoroquinolone antibacterial drugs in *Stenotrophomonas maltophilia* isolated from various departments of the hospital.

Department	No. Bacterial Isolate	Levofloxacin		Sulfamethoxazole		Minocycline	
		Resistant (%)	Susceptible (%)	Resistant (%)	Susceptible (%)	Resistant (%)	Susceptible (%)
ICU	113	2.7	97.3	72.1	27.9	0.9	98.2
Respiratory medicine	82	6.3	92.4	72.2	27.8	1.3	98.7
Neurosurgery	49	8.5	89.4	82.6	17.4	0	97.9
Pediatrics	43	0	97.7	72.1	27.9	0	100
Emergency	29	0	89.3	75	25	0	100
Neurology	20	0	95	95	5	0	100
Others	90	12.4	86.2	81.7	18.3	0	98.6

**Table 4:** Resistance of *Stenotrophomonas maltophilia* to three fluoroquinolone antibacterial drugs among patients at 3 age groups.

Age Group (years)	Bacterial Isolate		Levofloxacin		Sulfamethoxazole		Minocycline	
	No.	Percentage (%)	Resistant (%)	Susceptible (%)	Resistant (%)	Susceptible (%)	Resistant (%)	Susceptible (%)
<18	59	13.8	0	98.6	76.8	23.2	0	100
18–59	129	30.3	2.1	96.8	70.6	29.4	0.5	98.9
≥60	238	55.9	6.3	91.2	77.1	22.9	0.6	98.2

**Table 5:** *In vitro* susceptibility of *Stenotrophomonas maltophilia* to 7 antimicrobials (*n* = 114).

Antimicrobial	MIC_R_ (µg/mL)	MIC_50_ (µg/mL)	MIC_90_ (µg/mL)	Percentage of Susceptible Isolate (%)	Percentage of Intermediate Isolate (%)	Percentage of Resistant Isolate (%)
Norfloxacin	0.5–128	8	64	45.7	21.9	32.4
Ofloxacin	0.125–64	1	4	73.6	13.2	13.2
Chloramphenicol	0.25–64	8	64	51.7	23.7	24.6
Minocycline	0.25–64	1	4	90.3	4.4	5.3
Ceftazidime	0.5–128	8	128	66.7	10.5	22.8
Levofloxacin	0.25–64	2	8	73.7	14.0	12.3
Ciprofloxacin	0.25–64	1	4	54.4	23.7	21.9

MIC_R_, range of minimum inhibitory concentration (MIC); MIC_50_: MIC required to inhibit the growth of 50% of organisms; MIC_90_: MIC required to inhibit the growth of 90% of organisms.

**Table 6:** Effect of reserpine treatment on the susceptibility of 114 *Stenotrophomonas maltophilia* isolates to 3 fluoroquinolone antimicrobials.

Antimicrobial	MIC_R_ (µg/mL)	No. Resistant Isolate	Percentage of Resistant Isolate (%)	No. Intermediate Isolate	Percentage of Intermediate Isolate (%)	No. Susceptible Isolate	Percentage of Susceptible Isolate (%)
Norfloxacin	0.5–128	37	32.4	25	21.9	52	45.7
Norfloxacin +reserpine	0.5–128	25	21.9	19	16.7	70	61.4
Ciprofloxacin	0.25–64	25	21.9	27	23.7	62	54.4
Ciprofloxacin + reserpine	0.25–64	15	13.2	22	19.3	77	67.5
Ofloxacin	0.125–64	15	13.2	15	13.2	84	73.6
Ofloxacin + reserpine	0.125–64	11	9.6	11	9.6	92	80.8

**Table 7:** Comparison of fluoroquinolone drug resistance phenotype between efflux pump-positive and -negative isolates of *Stenotrophomonas maltophilia*.

Antimicrobial	Efflux Pump-Positive Isolate (*n* = 19)			Efflux Pump-Negative Isolate (*n* = 95)		
	Resistant (%)	Intermediate (%)	Susceptible (%)	Resistant (%)	Intermediate (%)	Susceptible (%)
Norfloxacin	18 (94.7)	0	1 (5.3)	19 (20)	25 (26.3)	51 (53.7)
Ciprofloxacin	16 (94.2)	1 (5.3)	2 (10.5)	9 (9.5)	26 (27.4)	60 (63.1)
Ofloxacin	15 (78.9)	3 (15.8)	1 (5.3)	0	12 (12.6)	83 (87.4)

### 3.4. Fluoroquinolone Resistance in S. maltophilia Isolated from Patients at Various Age Groups

No levofloxacin resistance was detected in the *S. maltophilia* isolated from patients aged <18 years, and the prevalence of levofloxacin resistance was higher in *S. maltophilia* isolated from patients aged ≥60 years than from patients aged <18 years (*p* = 0.046). A high prevalence of sulfamethoxazole resistance and low prevalence of minocycline resistance were observed in *S. maltophilia* clinical isolates (Table 4).

### 3.5. Effect of Reserpine on MIC of S. maltophilia to Fluoroquinolone

The MIC of norfloxacin, ofloxacin, chloramphenicol, minocycline, ceftazidime, levofloxacin, and ciprofloxacin on the 114 *S. maltophilia* clinical isolates from 2012 was shown in Table 5. All the 114clinical isolates of *S. maltophilia* grew well on the plate of M-H agar containing reserpine. There were two, 10 and 11 *S. maltophilia* clinical isolates with reduction in the MIC of norfloxacin, ciprofloxacin, and ofloxacin by 3/4 or more following reserpine treatment, respectively. These bacterial isolates were defined as pump positive; however, 3/4 or higher reduction in MIC of two fluoroquinolones was found in four isolates. Finally, a total of 19 efflux pump-positive *S. maltophilia* clinical isolates were identified. Our findings showed significant differences in the sensitivity of *S. maltophilia* to norfloxacin (χ^2^ = 4.788, *p* < 0.05) and ciprofloxacin (χ^2^ = 3.982, *p* < 0.05) before and after reserpine treatment, while no significant difference was detected in the susceptibility of *S. maltophilia* to ofloxacin (χ^2^ = 0.9, *p* > 0.05) (Table 6).

### 3.6. Association of Efflux Pump Phenotype with Resistance of S. maltophilia to Fluoroquinolone

The efflux pump-negative *S. maltophilia* isolates showed significantly greater susceptibility than efflux pump-negative isolates to norfloxacin, ciprofloxacin, and ofloxacin (χ^2^ = 7.326, 6.904 and 12.756, all *p* values < 0.05) (Table 7).

### 3.7. Prevalence of Fluoroquinolone Resistance Genes

*gyrA*, *parC*, *smeD*, *smeE* and *smeF* genes were detected in all 114 clinical isolates of *S. maltophilia*, with 100% prevalence seen, and the prevalence of *smqnr* gene was 25.4%; however, no other fluoroquinolone resistance genes were detected.

### 3.8. mRNA Expression of smeD and smeF in S. maltophilia

**Figure 1:**
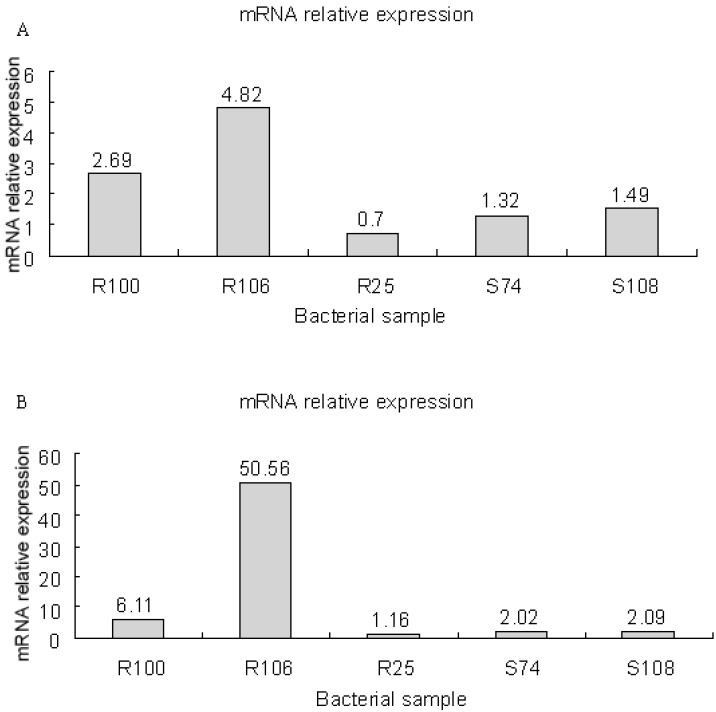
Relative mRNA expression of *smeD* (**A**) and *smeF* (**B**) genes.

High expression of *smeD* and *smeF mRNA* was detected in two efflux pump-positive, fluoroquinolone-resistant isolates of *S. maltophilia* (R100 and R106 isolates), while relatively low expression was observed in two efflux pump-negative, fluoroquinolone-susceptible isolates (S74 and S128 isolates) and one efflux pump-positive, fluoroquinolone-resistant isolate of *S. maltophilia* (R25) (Figure 1). The relative *mRNA* expression of *smeD* and *smeF* genes was 3.755 ± 1.506 and 28.335 ± 31.431 in efflux pump-positive *S. maltophilia*isolates, which was significantly higher than that (1.170 ± 0.416 and 1.757 ± 0.518) in efflux pump-negative isolates (*t* = 4.384 and 44.443, both *p* values < 0.05).

### 3.9. Sequence Analysis of gyrA, parC and smqnr Genes

*gyrA* (300 bp in length) and *parC* (273 bp in length) genes were detected in 95 efflux pump-negative isolates of *S. maltophilia*. The PCR products of the *gyrA* and *parC* genes extracted from five*S. Maltophilia* isolates were sequenced and then aligned with the sequence of the control ATCC13637 strain. Our findings showed Glu-Lys mutation (GAA-AAA) was detected at the 151th amino acid of the *gyrA* gene from two fluoroquinolone-resistant isolate of *S. maltophilia* (R101 and R138) and one fluoroquinolone-susceptible isolate (S62), while Gly-Arg mutation (GGC-CGC) was found at the 37th amino acid of the *parC* gene derived from two fluoroquinolone-resistant isolate of *S. maltophilia* (R101 and R138) and two fluoroquinolone-susceptible isolate (S31 and S126) (Table 8). There were no significant differences in the prevalence of mutation at 151th amino acid of the *gyrA* gene (*p* = 0.4) and at the 37th amino acid of the *parC* gene (*p* = 1) between the fluoroquinolone-susceptible and -resistant *S. maltophilia* isolates.

The *smqnr* gene was amplified from 14 randomly selected *S. maltophilia* isolates, sequenced, and aligned to the gene sequences of 58 *smqnr* variants accessed in GenBank. The sequence of the *smqnr* gene amplified from four isolates was completely consistent with the sequence of *smqnr11* gene (GenBank accession number: AB430848), that from three isolates completely consistent with the sequence of *smqnr35* gene (GenBank accession number: HQ896263), that from one isolate completely consistent with the sequence of *smqnr8* gene (GenBank accession number: AB430850), that from one isolate completely consistent with the sequence of *smqnr9* gene (GenBank accession number: AB430846), that from one isolate completely consistent with the sequence of *smqnr41* gene (GenBank accession number: HQ896273), and that from one isolate completely consistent with the sequence of *smqnr53* gene (GenBank accession number: AB852573). After sequence alignment between two fluoroquinolone resistant gene mutant isolates and *smqnr1* gene (GenBank accession number: AB430839), there were seven amino acid mutations found in *S. maltophilia* isolate 90, and 11 amino acid mutations found in *S. maltophilia* isolate 116. There were one or more differences in the amino acid sequence among the *smqnr* genes amplified from 14 *S. maltophilia* clinical isolates, with 92%–99% heterogenicity.

### 3.10. PFGE

**Figure 2:**
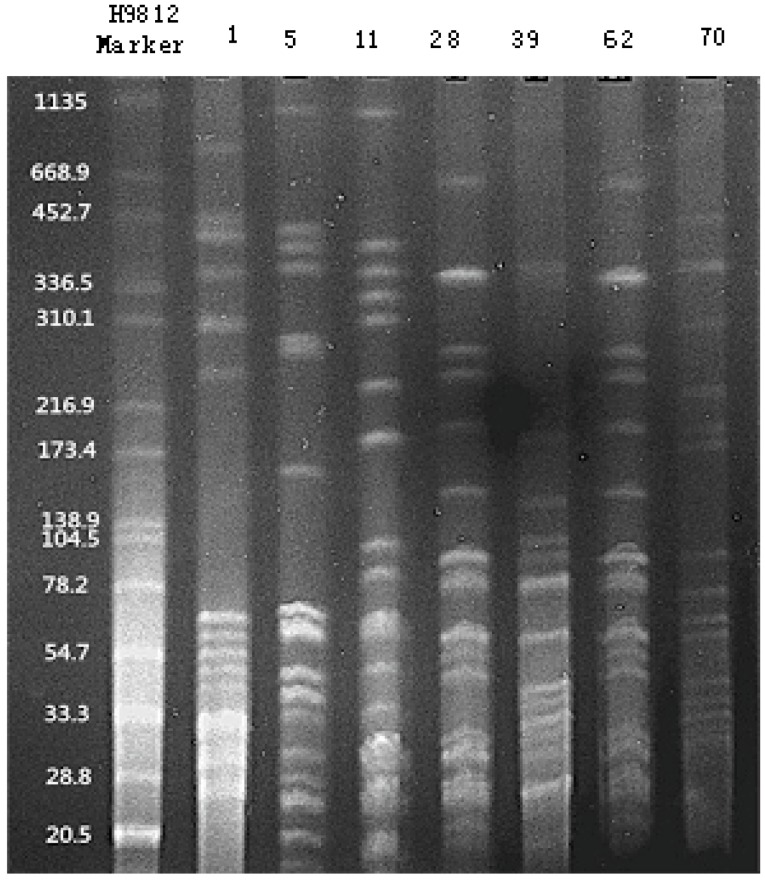
PFGE of 29 *smqnr* gene-positive *S. maltophilia* clinical isolates.

PFGE of 29 *smqnr* gene-positive *S. maltophilia* clinical isolates revealed 25 genotypes and 28 subgenotypes (Figure 2).

Isolates numbered 28 and 62 shared the same PFGE type, isolates numbered 70 and 91 showed two PFGE subgenotypes, with 1- to 3-band difference, and isolates 80, 138 and 141showed three PFGE subgenotypes, with 1- to 3-band difference.

## 4. Discussion

*S. maltophilia*, a non-fermentative bacterium, Gram-negative, conditionally pathogenic bacterium that frequently colonizes the human respiratory tract and intestinal tract, may induce pneumonia, meningitis and chronic enteritis [1,2,3].In this study, a total of 426 *S. maltophilia* strains were isolated from auniversity hospital in Northwestern China during the period from 2010 through 2012, which accounted for 10.1% (426/4,225) of total non-fermentative bacteria (it ranks third) and 1.8% (426/23,880) of total clinical bacterial isolates, and the prevalence of *S. maltophilia* ranked sixth among all Gram-negative bacilli. In addition, the majority of *S. maltophilia* strains were isolated from the respiratory tract specimens collected from the ICU, department of Respiratory Medicine and Department of Neurosurgery, and 55.9% of the *S. maltophilia* strains were isolated from patients aged over 60, suggesting that elderly patients are at high risk of *S. maltophilia* infections. Due to its inherent resistance to many antimicrobials, the antimicrobial susceptibility testing of *S. maltophilia* is of great importance to guide clinical antibiotic use [34]. Among the 426 *S. maltophilia* clinical isolates, the prevalence of levofloxacin, sulfamethoxazole and minocycline resistance was 3.3%, 74.3% and 0.5%, respectively, and a gradually increasing tendency was found in the prevalence of antimicrobial resistance in *S. maltophilia* year by year with the extensive use of antimicrobials.

**Table 8:** Amino acid mutation in *gyrA* and *parC* genes of 5*Stenotrophomonas maltophilia* isolates and MIC of three fluoroquinolone agents.

Bacteria Number	Antimicrobial MIC (µg/mL)			*gyrA*			*parC*	
	Norfloxacin	Ciprofloxacin	Ofloxacin	85th amino acid	119th amino acid	151th amino acid	37th amino acid	72th amino acid
ATCC13637	0.5	0.25	0.25	Valine (GTC)	Alanine (GCA)	Glutamic acid (GAA)	Glycin (GGC)	Glycin (GGT)
S31	4	0.5	0.5	Valine (GTG)	Alanine (GCG)	—	Arginine (CGC)	—
S62	2	1	1	Valine (GTG)	Alanine (GCG)	Lysine (AAA)	—	—
R101	32	8	16	Valine (GTG)	Alanine (GCG)	Lysine (AAA)	Arginine (CGC)	—
S126	8	2	2	Valine (GTG)	Alanine (GCG)	—	Arginine (CGC)	Glycin (GGC)
R138	32	4	8	Valine (GTG)	—	Lysine (AAA)	Arginine (CGC)	Glycin (GGC)

S31, S62 and S126 were fluoroquinolone-susceptible *Stenotrophomonas maltophilia* isolates; R101 and R138 were fluoroquinolone resistant.

The efflux pump inhibitor reserpine, an indole alkaloid, can block the substrate across the efflux channel, thereby increasing the intracellular drug concentrations [35]. Due to low specificity and affinity of reserpine-membrane glycoprotein binding, a high dose of these two drugs is required to achieve the inhibition; however, the dose that achieves inhibition on activity of efflux pump may induce neurotoxicity [36,37], which limits their clinical application. Our findings showed that reserpine inhibited the pumping out of fluoroquinolones in some *S. maltophilia* clinical isolates, resulting in a reduced prevalence of antimicrobial resistance. In this study, a total of 38 fluoroquinolone-resistant and 76 fluoroquinolone-susceptible *S. maltophilia* isolates were screened, and a significant difference was detected in the prevalence of efflux pump between the fluoroquinolone-resistant (18/38) and fluoroquinolone-susceptible bacterial isolates (1/76) (χ^2^ = 25.189, *p* < 0.05). These results demonstrated the efflux ability in both fluoroquinolone-resistant and -susceptible *S. maltophilia* isolates, and a higher activity was found in resistant isolates, indicating that efflux pump increases the resistance to *S. maltophilia* to fluoroquinolones. However, we did not identify *S. maltophilia* clinical isolates positive for efflux pump of all three fluoroquinolones, including norfloxacin, ciprofloxacin, and ofloxacin, indicating that fluoroquinolones have various efflux capabilities due to different biochemical characteristics caused by the structural difference.

Mutations of DNA gyrase and topoisomerase [15,16,17,18], mutation of drug resistance genes [19,20] and drug efflux pumps [21,22,23] are reported to be involved in the emergence of antibiotic resistance in *S. maltophilia.* Efflux pump system has been proved to be the most important cause responsible for the development of intrinsic and acquired multidrug resistance in *S. maltophilia* [20]. *SmeABC* multidrug efflux pump system is the first efflux pump gene identified in *S. maltophilia* [32], which is homogenous to the MexAB-OprM efflux pump system in *Pseudomonas aeruginosa* [38]. *SmeDEF*, a root nodulation and division (RND) family member, is an efflux pump system associated with multidrug resistance in *S. maltophilia* [39]. The substrates of *SmeDEF* include fluoroquinolones, macrolides and tetracyclines, which are mutually different in structures [40]. In the current study, we detected high mRNA expression of *smeD* and *smeF* genes in two efflux pump-positive, fluoroquinolone-resistant *S. maltophilia* isolates, and low expression in two efflux pump-negative, fluoroquinolone-susceptible isolates and one efflux pump-negative, fluoroquinolone-resistant isolate. These findings are consistent with those resulting from previous studies [41,42], indicating that *smeD* and *smeF* play vital roles in the development of resistance of *S. maltophilia* to fluoroquinolones.

Two enzymes, DNA gyrase and topoisomerase IV, are major targets of fluoroquinolones[43,44,45]. DNA gyrase consists of two subunits, GyrA and GyrB [46], and topoisomerase IV is composed of two ParC subunits (homogenous to GyrA) and two ParE subunits (homogenous to GyrB) [16]. In most gram-negative bacteria, *gyrA*is the primary target conferring fluoroquinolone resistance, *parC* as the secondary target, and the alteration of *gyrB* and *pare* expression is found to improve drug resistance level [47]. Therefore, *gyrA* and *parC* genes were amplified from three randomly selected fluoroquinolone-susceptible *S. maltophilia* clinical isolates and two randomly selected fluoroquinolone-resistant isolates and sequenced; however, we did not detect mutations associated with fluoroquinolone resistance that are frequently identified in other Gram-negative bacteria, such as mutations in the Ser-83 codons and Asp-87 codons of the *gyrA* gene and Gly-84 codons of the *parC* gene. In addition, there was significant difference found in the prevalence of *gyrA* and *parC* mutation between fluoroquinolone-susceptible and -resistant *S. maltophilia*, indicating that the mutation in the *gyrA* and *parC* genes did not correlate with fluoroquinolone resistance in *S. maltophilia*. Sequencing revealed almost identical nucleic acid sequences of *gyrA* and *parC* genes between fluoroquinolone-susceptible and -resistant isolates, which further demonstrated that the resistance of *S. maltophilia* to fluoroquinolones was not associated with targeted gene mutation. Such a finding was consistent with the results from the study Ribera *et al.* [48], but inconsistent with the study by Liu and colleagues [50]. Ribera and colleagues [48] reported no amino acid changes in the quinolone resistance-determining region (QRDR) of either *gyrA* or *parC* genes among quinolone-susceptible and -resistant *S. maltophilia* strains, and concluded that the development of resistance to quinolones was not related to mutations in the QRDR of *gyrA* and *parC* genes in the *S. maltophilia* isolates, contrary to what has been described in other microorganisms. Liu *et al.* [49] found an increase in the MIC of fluoroquinolones for *S. maltophilia* isolates with mutations in the *gyrA* or *parC* genes (8–16 μg/L) as compared to the isolates without mutations in the *gyrA* or *parC* genes (2–4 μg/L), and detected mutation at the 80th amino acid of the *parC* gene in one isolate, leading to substitution of serine by isoleucine (AGC-ATC), and at the 57th amino acid of the *gyrA* gene leading to substitution of alanine by arginine (GCG-CGC/CGG) and at the 44th amino acid of the *parC* gene (GGC-CGC) leading to substitution of glycine by arginine in two isolates, demonstrating that the mutation of *gyrA* and *parC* genes may be associated with the resistance to fluoroquinolone in *S. maltophilia*.

Plasmid-mediated quinolone resistance (PMQR) is a newly identified mechanism of antimicrobial resistance, and it is mainly mediated by antibiotic resistance *qnr* gene, including subtypes of *qnrA*, *qnrB*, *qnrC*, *qnrD* and *qnrS*, in which *qnrA* is the most common subtype [50]. PMQR is frequently detected in Enterobacteriaceae [51,52], and it has been detected in non-fermentative *P. aeruginosa* [53]. However, no PMQR is reported in *S. maltophilia* till now, and PMQR genes were not detected in the *S. maltophilia* clinical isolates identified in the present study.

It has been recently shown that *S. maltophilia* contains a chromosomally encoded *qnr* gene (*Smqnr*) which confers low-level resistance to fluoroquinolones [54]. *Smqnr* is a new quinolone resistance gene locating in the chromosome in *S. maltophilia*, which was firstly identified in 2008 [55]. There have been 58 *smqnr* genes identified so far, and only variations in several amino acids are found among these *smqnr* genes. In this study, the prevalence of *smqnr* gene was 25.4% in the 114 *S. maltophilia* clinical isolates included in this study.

Since *S. maltophilia* is considered a hospital-acquired colonizing bacterium, it does not receive much attention. However, *S. maltophilia* infections may cause high mortality. The spread of *S. maltophilia* isolates across a hospital would greatly threaten inpatients’ health. PFGE is accepted as the gold standard for genotyping bacteria due to high resolution and repeatability [56]. In this study, PFGE assay was employed for molecular identification of *S. maltophilia* clinical isolates, and 25 PFGE genotypes and 28 subgenotypes were detected in the 29 *S. maltophilia* clinical isolates; however, the predominant colony could not be identified due to the limited number of bacterial isolates with the same subgenotype.

## 5. Conclusions

*S. maltophilia* is an important hospital-acquired pathogenic bacterium, and sterilization and isolation of patients should be strengthened due to multidrug antimicrobial resistance and clonal spread of *S. maltophilia* clinical isolates. Monitoring the clinical distribution and antimicrobial resistance of *S. maltophilia* is of great significance for the clinical therapy of the bacterial infection. Reserpine is effective atinhibiting the active efflux of norfloxacin, ciprofloxacin and ofloxacinin *S. maltophilia* and reducing theMIC of fluoroquinolones against the bacteria. The resistance of *S. maltophilia* to fluoroquinolones shows no significant association with the alteration in the targeted site of DNA gyrase and topoisomerase IV, while the expression of efflux pump *smeD* and *smeF* genes correlates with the resistance of *S. maltophilia* to fluoroquinolones.
